# Lung mechanics and high-resolution computed tomography of the chest in very low birth weight premature infants

**DOI:** 10.1590/S1516-31802003000400006

**Published:** 2003-07-01

**Authors:** Rosane Reis de Mello, Maria Virgínia Peixoto Dutra, José Roberto Ramos, Pedro Daltro, Márcia Boechat, José Maria de Andrade Lopes

**Keywords:** Premature infant, Pulmonary function test, Lung compliance, Lung resistance, High resolution computed tomography, Prematuro, Prova de função pulmonar, Complacência pulmonar, Resistência pulmonar, Tomografia computadorizada de tórax de alta resolução

## Abstract

**CONTEXT:**

Premature infant lung development may be affected by lung injuries during the first few weeks of life. Lung injuries have been associated with changes in lung mechanics.

**OBJECTIVE:**

To evaluate an association between lung mechanics and lung structural alterations in very low birth weight infants (birth weight less than 1500 g).

**DESIGN:**

A cross-sectional evaluation of pulmonary mechanics (lung compliance and lung resistance) and high resolution computed tomography of the chest at the time of discharge, in 86 very low birth weight infants born at Instituto Fernandes Figueira, a tertiary public healthcare institution in Rio de Janeiro, Brazil. Lung compliance and resistance were measured during quiet sleep. High resolution computed tomography was performed using Pro Speed-S equipment.

**MAIN MEASUREMENTS:**

Statistical analysis was performed by means of variance analysis (ANOVA/ Kruskal Wallis). The significance level was set at 0.05.

**RESULTS:**

Abnormal values for both lung compliance and lung resistance were found in 34 babies (43%), whereas 20 (23.3%) had normal values for both lung compliance and lung resistance. The mean lung compliance and lung resistance for the group were respectively 1.30 ml/cm H_2_O/kg and 63.7 cm H_2_O/l/s. Lung alterations were found via high-resolution computed tomography in 62 (72%) infants. Most infants showed more than one abnormality, and these were described as ground glass opacity, parenchymal bands, atelectasis and bubble/cyst. The mean compliance values for infants with normal (1.49 ml/cm H_2_O/kg) high resolution computed tomography, 1 or 2 abnormalities (1.31 ml/cm H_2_O/kg) and 3 or more abnormalities (1.16 ml/cm H_2_O/kg) were significantly different (p = 0.015). Our data were insufficient to find any association between lung resistance and the number of alterations via high-resolution computed tomography.

**CONCLUSION:**

The results show high prevalence of lung functional and tomographic abnormalities in asymptomatic very low birth weight infants at the time of discharge. They also show an association between lung morphological and functional abnormalities.

## RESUMO

**CONTEXTO:**

O desenvolvimento pulmonar do bebê prematuro pode ser afetado por lesão ao pulmão nas primeiras semanas de vida, podendo ocasionar alterações na mecânica pulmonar.

**OBJETIVO:**

Avaliar a relação entre a mecânica pulmonar e as alterações estruturais pulmonares em prematuros de muito baixo peso (peso de nascimento inferior a 1.500 g).

**TIPO DE ESTUDO:**

Estudo de corte transversal avaliando a mecânica pulmonar (medidas a complacência e a resistência) e estrutura pulmonar (através da tomografia de tórax de alta resolução) próximo à alta hospitalar de 86 prematuros de muito baixo peso, nascidos no Instituto Fernandes Figueira, uma instituição pública, terciária, no Rio de Janeiro, Brasil. A complacência e resistência pulmonares foram medidas durante o sono tranqüilo. A tomografia de tórax foi realizada com o equipamento Pro Speed-S.

**MAIN MEASUREMENTS:**

Na análise estatística, utilizamos análise de variância para múltiplas comparações (ANOVA, Kruskal Wallis). O nível de significância estatística empregado foi 0,05.

**RESULTADOS:**

Em 34 bebês (43%), foram encontrados resultados alterados tanto para a complacência quanto para a resistência pulmonares e, em 20 crianças (23,3%), ambos os resultados foram normais. As médias da complacência e resistência pulmonares da população estudada foram respectivamente 1,30 ml/cm H2O/Kg e 63,7 cm H2O/l/s. Em 62 bebês (72%), os exames tomográficos apresentavam alterações pulmonares. A maioria das crianças mostrou mais de uma alteração, descritas como opacidade em vidro fosco, banda parenquimatosa, atelectasia, bolha/cisto. As médias dos valores de complacência para as crianças com tomografia de tórax normal, 1 ou 2 alterações e 3 ou mais alterações, foram significativamente diferentes: 1,49, 1,31, 1,16 (p = 0,015). Não encontramos relação entre resistência pulmonar e o número de alterações tomográficas.

**CONCLUSÃO:**

Os resultados mostram alta prevalência de anormalidades funcionais e tomográficas em bebês prematuros, assintomáticos, próximo à alta hospitalar. Mostram também uma relação entre anormalidades pulmonares morfológicas e funcionais.

## INTRODUCTION

The introduction of new drugs and treatment methods during the neonatal period has improved the prognosis for critically ill babies. The survival rate is now more than 70% for babies with birth weight between 700 and 900 g, and more than 90% for babies with birth weight greater than 1000 g.^1,2^ Nevertheless, this improvement may be accompanied by higher morbidity due to prematurity and/or intensive care sequelae in most organs and systems.

The lung is a particularly sensitive organ and serious lung injuries during the first few weeks of life can affect lung development.^3,4^ Koumbourlis et al.^5^ noted that postnatal lung injury in premature infants is associated with decreases in pulmonary volumes, changes in lung mechanics, airway obstruction and hyperinflation.

Very little research has been carried out for characterizing lung function^5,6,7,8^ and morphology^9,10^ in premature infants at the time of discharge. It is well known that chest x-rays are not sensitive enough to reveal lung parenchymal abnormalities and there is frequently a discrepancy between how the lung appears on an x-ray and the given respiratory symptoms.^11^ Although lung mechanics studies are reliable, they are not available on a clinical basis in most centers.

It has recently been shown that high resolution computed tomography can yield better results than conventional chest x-rays, thus suggesting that this may be an additional imaging technique to be used on infants with pulmonary disease.^12^

Up to the present day, we are unaware of any study showing an association between lung mechanical abnormalities and structural alterations of the lung parenchyma or airways in very low birth weight infants. The objective of this study was to conduct a pulmonary evaluation of very low birth weight infants by means of a pulmonary function test and high resolution computed tomography of the chest. We also aimed to demonstrate an association between lung mechanics and structural alterations of lung parenchyma in very low birth weight infants before hospital discharge.

## METHODS

We studied newborn infants of adequate development for their gestational age^13^ with birth weights of less than 1,500 g and gestational ages of less than 34 weeks, born between January 1998 and August 2000 at Instituto Fernandes Figueira (Fundação Oswaldo Cruz, Rio de Janeiro, Brazil), a tertiary public healthcare institution. Gestational age was calculated from the mother's last menstruation and confirmed by an obstetric ultrasound examination prior to the 20^th^ week of pregnancy. Babies with congenital malformations, infections and genetic syndromes, and those that were small for the gestational age, were excluded from this study.

The study was approved by the Research Ethics Committee of Instituto Fernandes Figueira and informed consent for the infants’ inclusion in the study was obtained from either one or both parents.

### Evaluation of lung function

Shortly before discharge from the nursery, a pulmonary function test was performed by a neonatologist who was unaware of the babies’ clinical history. The children were clinically stable and breathing room air. The pulmonary function test was carried out without sedation, with the baby in an incubator, in the supine position in quiet sleep (during non-REM sleep).

The following physiological signs were measured: flow, esophageal pressure and airway pressure. Airflow was measured using a face mask connected to a pneumotachograph (Fleisch 00) and an ultrasensitive pressure transducer (Validyne MP 45). Volume was given through the electronic integration of the flow signals collected. The flow signal was calibrated using a precision flowmeter. Esophageal pressure was measured using a water-filled catheter (Argyle 8) positioned in the lower third of the esophagus. The catheter was introduced through the oral cavity and an occlusion test was carried out to verify whether its positioning was adequate. This consisted of obstructing the airway at the end of the inspiration and checking whether the esophageal pressure was similar to the pressure in the mouth generated at the obstruction. The test is considered satisfactory when the alteration between occlusion and esophageal pressure is lower than 5%. When the pressures have equal results it means that there is no leak on the mask and the positioning of the catheter is correct. The catheter was connected to a Validyne DP 45 pressure transducer. Airway pressure was also measured using a Validyne DP 45 pressure transducer attached to the face mask.

All data were digitally input to a microcomputer through a 1280-A Data Translation Systems analog-digital board. The analog readings were interpreted by PCLAB software (Data Translations). The analysis and data processing were carried out using the Anadat/ Labdat software program (RHT Infodat, Montreal, Canada). The data collected were stored on a floppy disk in binary format for processing and analysis. Between 20 and 50 breathing cycles were selected per file. Lung resistance and lung compliance were calculated using linear regression analysis.^14^ In the linear regression technique (computer-based calculation) the description of the lung mechanical properties are derived from the general motion equation for the lung: P = (1/C x V) + (R x F) + (I x d^2^V/dt^2^), where P is the total pressure measured at any point of the respiratory cycle, i.e. the pressure developed by the respiratory muscles, C stands for compliance, V for volume, R for resistance, F for flow, I for inertance and d^2^V/dt^2^ for acceleration. The values for lung compliance were calculated and standardized for body weight.

Lung compliance values of less than 1.2 ml/ cm H_2_0/kg,^15^ and lung resistance greater than 50 cm H_2_O/l/s^16^ were defined as abnormal.

### Pulmonary structural evaluation via high resolution computed tomography of the chest

High resolution computed tomography was performed using Pro Speed-S (General Electric) equipment using high resolution slices of 90 mAs (60 mA for 1.5 seconds) and 120 kV. Each test consisted of 6 to 9 axial slices of 1 mm in thickness, at intervals of 10-15 mm. The tests were carried out without sedation, using the technique developed by Lucaya et al.^17^ (1996), which allows low doses of radiation without the loss of image quality. The tomographic images were analyzed independently by two radiologists who were unaware of any of the children's clinical histories. The inter-observer reliability for evaluating normal and abnormal tomographic images was assessed via Kappa statistics.^18^ Cases of disagreement between test results were solved by reaching a consensus between the specialists. Consequently, the data presented in this work is the result of agreement between two radiologists.

The parenchymal alterations visible via high resolution computed tomography were described as follows, according to the type and extent of the abnormality: a) aeration disturbance, b) parenchymal bands, c) atelectasis, d) consolidation, e) bronchial wall thickness, f) "ground-glass" opacity, g) interlobular septal thickening, h) subpleural opacity, or i) bubble/cyst.^19^

We defined bronchopulmonary dysplasia (BPD) as oxygen dependence beyond the 28^th^ day of life, in association with chest x-ray abnormalities.^20^

## DATA ANALYSIS

The compliance and resistance values for each group of tomographic alterations were considered to be the outcome variable and the comparisons were performed via variance analysis. ANOVA, the variance analysis for comparisons between more than two means, was used in cases of homogeneous variance and normal distribution of samples, and Kruskal/Wallis variance analysis was used in cases of unequal variance. p values of less than 0.05 were taken to indicate significant differences between groups.

Data were analyzed by using the STATA Intercooled program (Stata Corporation, College Station, TX).

## RESULTS

A total of 179 babies with birth weight of less than 1,500 g and gestational age of less than 34 weeks were admitted to the Neonatology Department between January 1998 and August 2000. Of these, 20 (11.2%) died during the hospitalization period and, in 4 cases, the parents refused to participate in the study. A total of 58 babies were excluded from the study, for the following reasons: 41 (22.9%) were small for the gestational age, 7 (3.9%) had genetic syndromes, 7 (3.9%) congenital malformations and 3 congenital infections (1.6%). Ninety-seven children satisfied the inclusion criteria for the study. Of these, 11 did not undergo computed tomography of the chest and were therefore not included in the study. Hence, the study was carried out using a sample of 86 children. Sixty-three mothers (73.3%) received antenatal steroids. The characteristics of the subjects included are described in Table 1. The neonatal morbidity encountered is shown in Table 2.

**Table 1:** Characteristics of the study group, formed by babies born in Instituto Fernandes Figueira, from 1998 to 2000

Birth weight (g)	1,101 (235.8)*
Gestational age (weeks)	28 (2.3)*
Apgar score at 5 min	8 ‡
Duration of neonatal unit stay (days)	59 (26.6)*
Male sex - n (%)	43 (50.0)
Cesarean section delivery - n (%)	45 (52.3)
Birth weight < 1,000 g - n (%)	27 (31.3)
Gestational age < 32 weeks - n (%)	67 (89.3)
Oxygen dependency (hours)	149 ‡

* *mean ± (standard deviation)*

‡ *median*

**Table 2:** Neonatal morbidity of very low birth weight infants who had adequate weight for their gestational age, born in Instituto Fernandes Figueira, from 1998 to 2000

Neonatal morbidity	n (%)
Apnea	60 (69.8)
Respiratory distress syndrome	39 (45.3)
Need for mechanical ventilation	39 (45.3)
Need for surfactant therapy	28 (32.6)
Bronchopulmonary dysplasia	24 (28.0)

At the time of the pulmonary function test, the mean weight was 1846 ± 451 g and the corrected gestational age, 36.0 ± 2 weeks. The mean value for lung compliance was 1.30 ± 0.44 ml/cm H_2_O/kg and for resistance, 63.7 ± 24.87 cm H_2_O/l/s. Only 20 of all the tests undertaken (n = 86) showed normal values for compliance and resistance: 39 (45.3%) had compliance/kg of less than 1.2 ml/cm H_2_O/ Kg and 57 (69.5%) had resistance of greater than 50 cm H_2_O/l/s. In 34 babies (43%), the values for compliance and resistance were abnormal according to our criteria.

A total of 24 children (28%) were classified as having bronchopulmonary dysplasia, and the mean lung compliance value in this group was 1.0 ± 0.35 ml/cm H_2_O/kg (95% confidence interval, CI of 0.88-1.12). For the infants without bronchopulmonary dysplasia, the mean value was 1.41 ± 0.42 ml/cm H_2_O/ kg (95% CI of 1.35-1.47; p < 0.05). The lung resistance was 70.04 ± 28.26 cm H_2_O/l/s (95% CI of 60.38-79.70) in bronchopulmonary dysplasia infants and 61.20 ± 23.20 cm H_2_O/ml/s (95% CI of 58.13-64.27) in those without bronchopulmonary dysplasia.

The inter-observer reliability for evaluating normal and abnormal high resolution computed tomography was 0.71 (measured via Kappa statistics). Among the 86 high resolution computed tomography scans, only 24 (27.9%) were considered normal. Aeration disturbance occurred in 33 cases (38.4%), parenchymal band in 30 (34.9%), atelectasis in 27 (31.4%), "ground-glass" opacity in 37 (43.0%), thickening of interlobular septa in 16 (18.6%), subpleural opacity in 18 (20.9%) and bubbles/cyst in 8 (9.3%). Most infants presented more than one abnormality (Table 3).

**Table 3:** High-resolution computerized tomography: number of abnormalities among very low birth weight infants who had adequate weight for their gestational age before discharge, born in Instituto Fernandes Figueira, from 1998 to 2000

number of abnormalities	number of patients (%)
None	24 (27.9)
1 or 2	28 (32.6)
≥ 3	34 (39.5)

The mean values of lung compliance for the groups with normal tomography, 1 or 2 alterations and 3 or more alterations were respectively 1.49 ± 0.40 ml/cm H_2_O/kg (95% CI of 1.35-1.63), 1.31 ± 0.46 (95% CI of 1.17-1.45) and 1.16 ± 0.42 (95% CI of 1.051.27). The variance analysis performed on the lung compliance values had a statistically significant result (p = 0.015).

The mean lung resistance values for the groups with normal tomography, 1 or 2 alterations and 3 or more alterations were respectively 60.60 ± 25.87 cm H_2_O/l/s (95% CI of 51.76-69.44), 60.65 ± 19.07 cm H_2_O/l/s (95% CI of 58.82-66.48) and 68.34 ± 28.15 cm H_2_O/l/s (95% CI of 60.94-75.74). The variance analyses for lung resistance values among the three groups of tomographic alterations were not statistically significant (Table 4).

**Table 4:** High-resolution chest tomography versus. lung compliance and resistance, for very low birth weight infants who had adequate weight for their gestational age, born in Instituto Fernandes Figueira, from 1998 to 2000

	HRCT Normal (n = 24)	HRCT 1 or 2 abnormalities (n = 28)	HRCT ≥ 3 abnormalities (n = 34)
Lung compliance per kg	1.49 ± 0.40*	1.31 ± 0.46*	1.16 ± 0.42*
± SD			
(95% CI)	(1.35-1.63)	(1.17-1.45)	(1.05-1.27)
Lung Resistance	60.60 ± 25.87	60.65 ± 19.07	68.34 ± 28.15
± SD			
(95% CI)	(51.76-69.44)	(58.82-66.48)	(60.94-75.74)

* *p = 0.015; HRCT = high resolution computerized tomography; CI = confidence interval; SD = standard deviation*

We were able to verify an association between lung compliance and several tomographic abnormalities.

## DISCUSSION

This was a prospective study undertaken in a neonatal intensive care unit with premature very low birth weight infants. The clinical data from these babies showed moderate severity among this group of infants, with 31% of the babies having birth weights of less than 1,000 g and about 45% requiring mechanical ventilation. The incidence of bronchopulmonary dysplasia was 28%, defined as the use of oxygen beyond 28 days of life.

Various techniques can be used for measuring lung compliance and lung resistance in newborns. Among the commonly used techniques are multiple occlusion, the conventional (Mead-Whittenberger) method and linear regression. In this study, the latter was used. The conventional and linear regression techniques measure lung compliance and resistance, whereas the multiple occlusion technique measures compliance and resistance of the respiratory system, which includes measurements of lung and thoracic wall mechanics. Both the conventional and linear regression techniques have the advantage of making the measurement during spontaneous breathing, without the need for the respiratory muscles to relax.^21^ The multiple occlusion and conventional techniques have the limitation of needing to assume a linear relation between pressure and volume. However, several authors have already described a non-linear relationship between pressure and volume in newborns with pulmonary diseases.^22^ Moreover, the high compliance of the thoracic wall observed in extremely premature infants (birth weight less than 1,000 g) may also alter the linearity of the pressure/volume relationship, since it can cause uneven distribution of pleural pressure and inadequate air filling of the lungs.

In the linear regression technique, the analysis is done by computer and calculation of the compliance and resistance is done at several points in the respiratory cycle (not only two or three, as in the conventional technique), which gives rise to high precision in the results and diminishes the influence of possible artifacts. Computer-based techniques allow data to be collected for extended periods of time and the distribution of various points on the regression curve to be observed. Thus, identification of non-linear points allows their exclusion and respiratory mechanics can then be calculated. The conventional and multiple occlusion techniques require many manual calculations for the assessment of pulmonary mechanics of the newborn, and they are slow and arduous. Authors who have compared pulmonary compliance and resistance measured by the conventional and linear regression techniques have found excellent correlation coefficients.^22,23^

We found a high prevalence of abnormalities in the pulmonary function test and high resolution computed tomography, which probably reflects lung injury caused by exposure of the immature lung to oxygen and mechanical ventilation. About 72% of the babies had some tomographic alteration and 45% displayed altered lung compliance.

The mean values of lung compliance (1.30 ± 0.44 ml/cm H_2_O/kg) and lung resistance (63.7 ± 24.87 cm H_2_O/l/s) in our sample are in agreement with the literature. Zaballos et al.^24^ studied the lung function of 18 very low birth weight premature infants (average weight of 1,288 ± 196 g and gestational age of 30.1 ± 2.7 weeks) and found that, for children of more than 7 days old, the mean compliance and resistance values were respectively 1.31 ± 0.3 ml/cm H_2_O/kg and 61.3 ± 36.4 cm H_2_O/ l/s, which are comparable with our results. Abbasi and Bhutani^16^ studied the lung mechanics of very low birth weight infants with gestational ages of less than 34 weeks, who did not need mechanical ventilation, and found values of 1.4 ± 0.1 for compliance and 49.0 ± 6.0 for resistance. In our laboratory, Viviani^21^ studied the lung function of 13 premature infants without respiratory disease in the neonatal period, with postnatal ages ranging from 28 to 47 days and gestational ages of between 32 and 34 weeks, and found mean values of lung compliance and resistance of respectively 1.55 ± 0.6 ml/cm H_2_O/kg and 53.6 ± 16.7 cm H_2_O/l/s.

Our data also show a significant association between high resolution computed tomography alterations and decreased lung compliance (Table 4). In most cases, the babies with more than three abnormalities seen via computed tomography used more mechanical ventilation and oxygen, and developed chronic lung disease. Studies in newborn animals have shown that hyperoxia and barotrauma can cause morphological and functional alterations in the lung. Warner et al.^25^ showed that prolonged neonatal hyperoxia in mice caused a decrease in alveolar septation, an increase in the size of terminal airways, an increase in lung fibrosis and an increase in the number of inflammatory cells in the lung. Hyperoxia also caused functional alterations with a decrease in lung volume and compliance. Davis et al.^26^ concluded that, 48 hours after being submitted to hyperoxia and hyperventilation, animals presented decreased dynamic lung compliance and increased lung resistance. Moderate-toserious atelectasis, exsudate fibrosis, edema and inflammation were all identified in these animals via microscopy.

Our tomographic findings seem to reflect these microscopic alterations described in animals. Compromised interstices can therefore affect lung distensibility and, consequently, the compliance. Decreased lung compliance can also be explained by peribronchial fibrosis, substitution of normal lung tissue by fibrotic parenchymal bands^27^ and decreased numbers of alveoli due to lung injury.^4^ The relationship between high resolution computed tomography and compliance in our data indicates primarily parenchymal disease.

The lack of an association between pulmonary resistance and lung morphology can be due to the following factors:

- The disease in these premature infants mainly involves the pulmonary paren chyma and not so much the airways. Recent articles have described altered lung anatomy in newborns who died of bronchopulmonary dysplasia due to factors that were present in the pre-surfactant era such as severe fibrosis, airway injury, hyperplasia and lung inflammation, which mainly showed as decreases in alveolar septation and vascular development.^28^
- There may have been minor alterations in the airways that were not evident from the high-resolution computed tomography.
- The test method used consisted of a series of images during spontaneous respiration, given that the patients in this study had very low weight and were breathing room air, thus not justifying the use of a more aggressive method such as tracheal intubation, which would have allowed the test to be done in two phases: inspiratory and expiratory apnea. This method of a series of inspirations and expirations with apnea is especially known as a means for detecting the presence of injuries that suggest that the small airways are compromised.^29^

Very few studies have been published using high resolution computed chest tomography on babies, but none has attempted to correlate these findings with functional lung tests in very low birth weight infants. Papers published previously involving high resolution computed tomography on premature infants basically focused on babies who developed bronchopulmonary dysplasia and were evaluated when they were close to or over one year old, and therefore utilized methodologies quite different from ours. Oppenheim et al.^12^ analyzed high resolution computed tomography from 23 children with bronchopulmonary dysplasia and an average age of 4 years, and found tomographic abnormalities in all the children. The alterations included multifocal areas of hyperaeration and well-defined linear and subpleural opacities. In 20 of the 23 children the three types of tomographic alterations were concurrent. Lee et al.^30^ evaluated the tomographic results from 6 children with bronchopulmonary dysplasia and an average age of 4 months, and found bubbles and focal emphysema in three patients and atelectasis in three others. Although these studies were carried out on older children, the abnormalities described, including bubbles, opacities, and hyperaeration, were similar to those shown in our study.

In summary, the results reported in this paper show that there is high prevalence of functional and tomographic alterations among asymptomatic very low birth weight infants at the time of discharge from the nursery, and that there is an association between lung morphological and functional abnormalities.

## References

[1] Hack M, Wright LL, Shankaran S (1995). Very-low-birth-weight outcomes of the National Institute of Child Health and Human Development Neonatal Network, November 1989 to October 1990. Am J Obstet Gynecol.

[2] Cooper TR, Berseth CL, Adams JM, Weisman LE (1998). Actuarial survival in the premature infant less than 30 weeks’ gestation. Pediatrics.

[3] Hakulinen AL, Heinonen K, Lansimies E, Kiekara O. (1990). Pulmonary function and respiratory morbidity in school-age children born prematurely and ventilated for neonatal respiratory insufficiency. Pediatr Pulmonol.

[4] Jobe AH, Ikegami M (1998). Mechanisms initiating lung injury in the preterm. Early Hum Dev.

[5] Koumbourlis AC, Motoyama EK, Mutich RL, Mallory GB, Walczak SA, Fertal K (1996). Longitudinal follow-up of lung function from childhood to adolescence in prematurely born patients with neonatal chronic lung disease. Pediatr Pulmonol.

[6] Gerhard T, Reifenberg L, Duara S, Bancalari E (1989). Comparison of dynamic and static measurements of respiratory mechanics in infants. J Pediatr.

[7] Chan KN, Wong YC, Silverman M (1990). Relationship between infant lung mechanics and childhood lung function in children of very low birthweight. Pediatr Pulmonol.

[8] Fitzgerald DA, Mesiano G, Brosseau L, Davis GM (2000). Pulmonary outcome in extremely low birth weight infants. Pediatrics.

[9] Moya MP, Bisset GS, Auten RL, Miller C, Hollingworth C, Frush DP (2001). Reliability of CXR for the diagnosis of bronchopulmonary dysplasia. Pediatr Radiol.

[10] Yuksel B, Greenough A, Karani J, Page A (1991). Chest radiograph scoring system for use in pre-term infants. Br J Radiol.

[11] Northway WH, Moss RB, Carlisle KB (1990). Late pulmonary sequelae of bronchopulmonary dysplasia. N Engl J Med.

[12] Oppenheim C, Mamou-Mani T, Sayegh N, Blic J de, Scheinmann P, Lallemand D (1994). Bronchopulmonary dysplasia: value of CT in identifying pulmonary sequelae. AJR Am J Roentgenol.

[13] Lubchenco LO, Hansman C, Dressler M, Boyd E (1963). Intrauterine growth as estimated from liveborn birth-weight data at 24 to 42 weeks gestation. Pediatrics.

[14] Greenspan JS, Abbasi S, Bhutani VK (1988). Sequential changes in pulmonary mechanics in the very low birth weight (less than or equal to 1000 grams) infant. J Pediatr.

[15] (1993). Respiratory mechanics in infants: physiological evaluation in health and disease. American Thoracic Society/European Respiratory Society. Am Rev Respir Dis.

[16] Abbasi S, Bhutani VK (1990). Pulmonary mechanics and energetics of normal, non-ventilated low birthweight infants. Pediatr Pulmonol.

[17] Lucaya J, Garcia-Pena P, Souza P, Sotil J (1996). Low-dose and special chest CT techniques. Pediatr Radiol.

[18] Fleiss JL (1981). Statistical methods for rates and proportions.

[19] Webb WR, Müller NL, Naidich DP, Webb WR, Muller NL, Naidich DP (1996). HRCT findings of lung disease. HighResolution CT of the lung.

[20] Bancalari E, Abdenour GE, Feller R, Gannon J (1979). Bronchopulmonary dysplasia: clinical presentation. J Pediatr.

[21] Viviani AR (1997). Avaliação do desenvolvimento funcional pulmonar dos recém-nascidos pretermo.

[22] Ramos JRM (1994). Estudo da complacência e respiratório do recémnascido a termo.

[23] Silva G, Gerhardt T, Silberg A, Gerhardt T, Claure N, Duara S, Bancalari E (1992). Nonlinear pressure/volume relationship and measurement of lung mechanics in infants. Pediatr Pulmonol.

[24] Benito Zaballos MF, Pedraz García C, Salazar A-Villalobos V (1991). Función pulmonar en recién nacidos pretérmino y a término durante el periodo neonatal: I. patrón respiratorio. [Pulmonary function in preterm and full term infants during the neonatal period: 1. respiratory pattern]. An Esp Pediatr.

[25] Warner BB, Stuart LA, Papes RA, Wispé JR (1998). Functional and pathological effects of prolonged hyperoxia in neonatal mice. Am J Physiol.

[26] Davis JM, Dickerson B, Metlay L, Penney DP (1991). Differential effects of oxygen and barotrauma on lung injury in the neonatal piglet. Pediatr Pulmonol.

[27] Van Lierde S, Smith J, Devlieger H, Eggermont E (1994). Pulmonary mechanics during respiratory distress syndrome in the prediction of outcome and differentiation of mild and severe bronchopulmonary dysplasia. Pediatr Pulmonol.

[28] Coalson JJ, Bland RD, Coalson JJ (2000). Pathology of chronic lung disease of early infancy. Chronic lung disease in early infancy.

[29] Siegel MJ, Siegel MJ (1999). Lung, pleura and chest wall. Pediatric body CT.

[30] Lee H, Price A, Rosenfelt W (1992). Increased detection of pathologic lung changes in infants with bronchopulmonary dysplasia (BPD) using chest computerized tomography (CT) [abstract]. Ped Res.

